# Virtual Reality and Sound Intervention under Chemotherapy (ViSu): study protocol for a three-arm randomised-controlled trial

**DOI:** 10.1136/bmjopen-2024-094040

**Published:** 2025-04-09

**Authors:** Steffen Holsteg, Luisa Ernsten, Nora K Schaal, Luisa M Keßling, Nele Schmutzler, Tanja N Fehm, Verena Friebe, Norbert Gattermann, Eugen Ruckhäberle, André Karger

**Affiliations:** 1 Center for Integrated Oncology Düsseldorf (CIO-D), Düsseldorf, Germany; 2 Clinical Institute for Psychosomatic Medicine and Psychotherapy, Medical Faculty & University Hospital Düsseldorf, Heinrich-Heine-Universitat Düsseldorf, Düsseldorf, Germany; 3 Department of Experimental Psychology, Heinrich Heine University Düsseldorf, Düsseldorf, Germany; 4 Department of Gynecology and Obstetrics, University Hospital Düsseldorf, Heinrich Heine University Düsseldorf, Düsseldorf, Germany; 5 Department of Hematology, Oncology, and Clinical Immunology, Medical Faculty and University Hospital Düsseldorf, Heinrich-Heine-Universitat Düsseldorf, Düsseldorf, Germany

**Keywords:** Virtual Reality, Mindfulness, CHEMOTHERAPY, eHealth, Stress, Psychological

## Abstract

**Introduction:**

Patients undergoing chemotherapy often experience side effects during treatment, including psychological distress and symptoms of anxiety and depression. Interventions during chemotherapy that divert attention from potentially aversive environmental factors have been demonstrated to have a beneficial impact on these symptoms. Virtual reality (VR) offers the potential to visually and audibly disengage from the surrounding environment and can create an alternative sense of presence. This could facilitate the implementation of active guided interventions that may prove more effective than receptive interventions, such as listening to music. The present study examines the feasibility, acceptance and effectiveness of a VR intervention and a music intervention during chemotherapy.

**Methods:**

The single-centre three-arm, randomised-controlled trial investigates the efficacy of a VR mindfulness intervention and a music intervention in patients with cancer undergoing chemotherapy at the University Hospital Düsseldorf, Germany. Patients were randomly assigned to receive either (a) the VR mindfulness intervention, (b) the receptive music intervention or (c) the standard care (control group) in two consecutive chemotherapy sessions. A comprehensive psychological assessment and self-ratings using visual analogue scales will be conducted with situational anxiety as the primary outcome measure. Additionally, secondary measures will be employed to assess cancer-related anxiety, self-efficacy and chemotherapy-related side effects. Furthermore, salivary cortisol, heart rate and blood pressure will be recorded. At the end of the study, an evaluation questionnaire will be completed. It is planned to enrol 82 patients.

**Ethics and dissemination:**

The study has been approved by the ethics committee of the medical faculty of the Heinrich-Heine-University Düsseldorf (2022-1880). Written informed consent is obtained from the patients prior to participation. The results will be published in international scientific, peer-reviewed journals. Conference presentations are also planned.

**Trial registration number:**

German Clinical Trials Register (DRKS00029738).

**STRENGTHS AND LIMITATIONS OF THIS STUDY**

- Randomised controlled trial that compares the effectiveness of two interventions (virtual reality mindfulness and music) to a control group.
- Assessment of a clinical population undergoing active oncological treatment.
- In addition to psychological assessments and self-ratings, saliva samples used to measure cortisol levels, heart rate and blood pressure are evaluated and provide a more objective method.
- Limited standardisation of the assessment environment, making it challenging to control for potential confounding variables during data collection.

## Introduction

 Although chemotherapy is an essential part of treatment and a potential condition for healing, many patients in oncology experience chemotherapy as distressing and time-consuming, taking place in an unpleasant surrounding. Chemotherapy cycles are frequently accompanied by adverse side effects such as nausea, fatigue or symptoms of anxiety and depression.^1^ Negative psychological side effects can have an additional negative impact on health-related quality of life^2 3^ or even reduced treatment adherence,^4 5^ which is associated with a poorer overall prognosis.^6 7^

Psychological interventions that are already offered during chemotherapy sessions have the potential to alleviate distress and mitigate side effects, thereby helping patients cope with the unpleasant environment. These interventions can be broadly categorised into receptive and active types. Receptive interventions, such as listening to music, have been shown to reduce anxiety, depressive symptoms and distress during chemotherapy.^8 9^ Active interventions like progressive muscle relaxation^10^ guided imagery^11^ or mindfulness-based interventions^12^ showed reducing effects on several psychological outcomes like anxiety, depression and distress. Additionally, active interventions may enhance patients’ self-efficacy during their whole cancer treatment.^13^ However, these interventions often require some instructions and guidance from a third person, an active participating role from the patient, and some level of training. The unpleasant context during chemotherapy, characterised by shared treatment rooms, noise and variability in treatment duration, offers various sources of distraction that can prevent patients from successfully performing or concentrating on the intervention.

Virtual reality (VR) offers a novel approach to supportive psycho-oncological interventions and could be beneficial as the feelings of presence and immersion could help patients to focus on the task by shielding them from distracting environmental factors. VR has been perceived as helpful during mindfulness training,^14^ and research suggests that VR-based mindfulness training may be superior to traditional mindfulness practices in reducing anxiety, stress, depressive symptoms, mood disturbances and sleep problems.^15^ For oncology patients undergoing chemotherapy, VR has been used primarily as a passive distraction intervention, proving more effective than other commonly used interventions, such as music.^16^ Furthermore, an observational study has reported that VR-based active interventions incorporating mindfulness practice can have positive effects on psychological side effects.^17^

Despite promising findings, the use of VR in chemotherapy remains underexplored, particularly as an active intervention. A recent systematic review and meta-analysis of VR use in patients with cancer receiving chemotherapy reported twelve randomised controlled trials (RCTs),^18^ all of them using VR as a passive distraction intervention. This highlights a significant gap in understanding the potential benefits of immersive, active VR interventions compared with passive approaches. Additionally, little is known about the factors that influence the feasibility of implementing such active or passive VR-based interventions in a clinical setting, which is particularly important given the technical challenges associated with VR setup compared with more routine interventions like music therapy.

Therefore, the Virtual Reality and Sound Intervention under Chemotherapy (ViSu) study aimed to investigate the effects of an active VR-based mindfulness intervention compared with a receptive music intervention and a control group on both subjective and objective psychological outcomes during two consecutive chemotherapy sessions. Given that active VR-based interventions may offer advantages over receptive music interventions due to the enhanced immersion and presence they provide, which may increase patient engagement, we hypothesise that the effects will be more pronounced in the VR group compared with the music group. Nonetheless, both interventions are expected to be superior to the control group, that receives only standard care. Furthermore, this study aims to identify key factors influencing the feasibility of implementing VR and music-based interventions in an outpatient chemotherapy setting, contributing to the growing body of evidence on supportive care in oncology.

## Methods

### Study design

The ViSu study is a single-centre, three-arm, non-blinded, randomised, controlled trial designed to investigate the efficacy of a VR mindfulness intervention and a music intervention in patients with cancer undergoing chemotherapy at the Interdisciplinary Outpatient Chemotherapy Center (IAC), University Hospital Düsseldorf, Germany. Participants will be randomly assigned to one of three study arms: (a) an intervention group receiving a VR-based mindfulness task, (b) an intervention group listening to music and (c) a control group with no intervention. The study protocol was developed in accordance with the Standard Protocol Items: Recommendations for Interventional Trials reporting guidelines.^19^ The checklist can be found in online supplemental appendix A.

The primary objective is to compare the effectiveness of the two interventions against a control group in reducing patients’ anxiety during two consecutive chemotherapy sessions. To evaluate whether the active VR intervention leads to any training or habituation effects, each participant will take part in two intervention sessions. Data collection will be conducted at both time points. Due to individual differences in chemotherapy schedules, the exact duration of chemotherapy treatment may differ between participants.

### Sample

Patients are either referred to the study by their attending physician during their consultation hours, recruited by the study team at the IAC, or contacted by the study team independently. For patients recruited by the study team or who self-refer, the study team will obtain the consent of the attending physician. All patients are prescreened for eligibility by their attending physician. The eligibility criteria for the study include (a) the general physical condition of the patient, (b) an age of 18 years or older, (c) the presence of a gynaecological (C51–55), senological (C50), haematological (C81–85, C91–95) or gastrointestinal cancer (C15ff, C16ff, C18ff, C20ff, C22, C23, C25ff) diagnosis according to ICD-10, (d) a chemotherapy duration of at least 60 min, (e) the presence of at least five remaining consecutive chemotherapy sessions, (f) sufficient knowledge of the German language and (g) at least moderate anxiety as measured by the Generalized Anxiety Disorder-7 (GAD-7) questionnaire.^20 21^

Patients are not eligible for the study if they have (a) severe visual and/or hearing impairment (self-reported), (b) brain metastases, (c) pre-existing neurological and/or psychiatric conditions affecting the vestibular system, impair balance or alter visual perception, (d) epilepsy, (e) claustrophobia, (f) severe side effects after the first chemotherapy session or (g) wearing cooling gloves or a cooling cap during the chemotherapy sessions.

Patients can withdraw from the study at any time. Participation will be terminated if chemotherapy sessions have to be interrupted due to the patient’s physical weakness or if chemotherapy is interrupted due to technical malfunctions. Participation will also be terminated if acute side effects or allergic reactions occur during chemotherapy, or if the oncological disease progresses negatively during ongoing therapy.

### Interventions

#### VR mindfulness intervention

Mindfulness, as defined by Kabat-Zinn,^22^ refers to the intentional self-regulation of attention to the present moment without judgement. To cultivate mindfulness skills, these practices are typically embedded in meditation exercises.^22^ Over time, this concept has been integrated into psychological interventions and has evolved into a variety of meditation practices, many of which have been tested in RCTs.^23^ Such exercises involve directing attention to thoughts, emotions and bodily sensations, simply observing them as they arise and pass away.^24^ Potential mechanisms underlying the effects of mindfulness include attention and emotion regulation, increased body awareness, and a shift in perspective on the self.^24^ VR may offer several advantages for mindfulness meditation. By shielding users from distracting environmental factors that might otherwise interfere with meditation, VR can create a more focused and immersive experience.^14 15^ The sense of presence that arises during VR exercises is described as engaging and is considered to enhance mindfulness.^14^ The combination of audiovisual stimuli in VR may further reduce mind-wandering by anchoring attention.^14 25^

**Figure 1:**
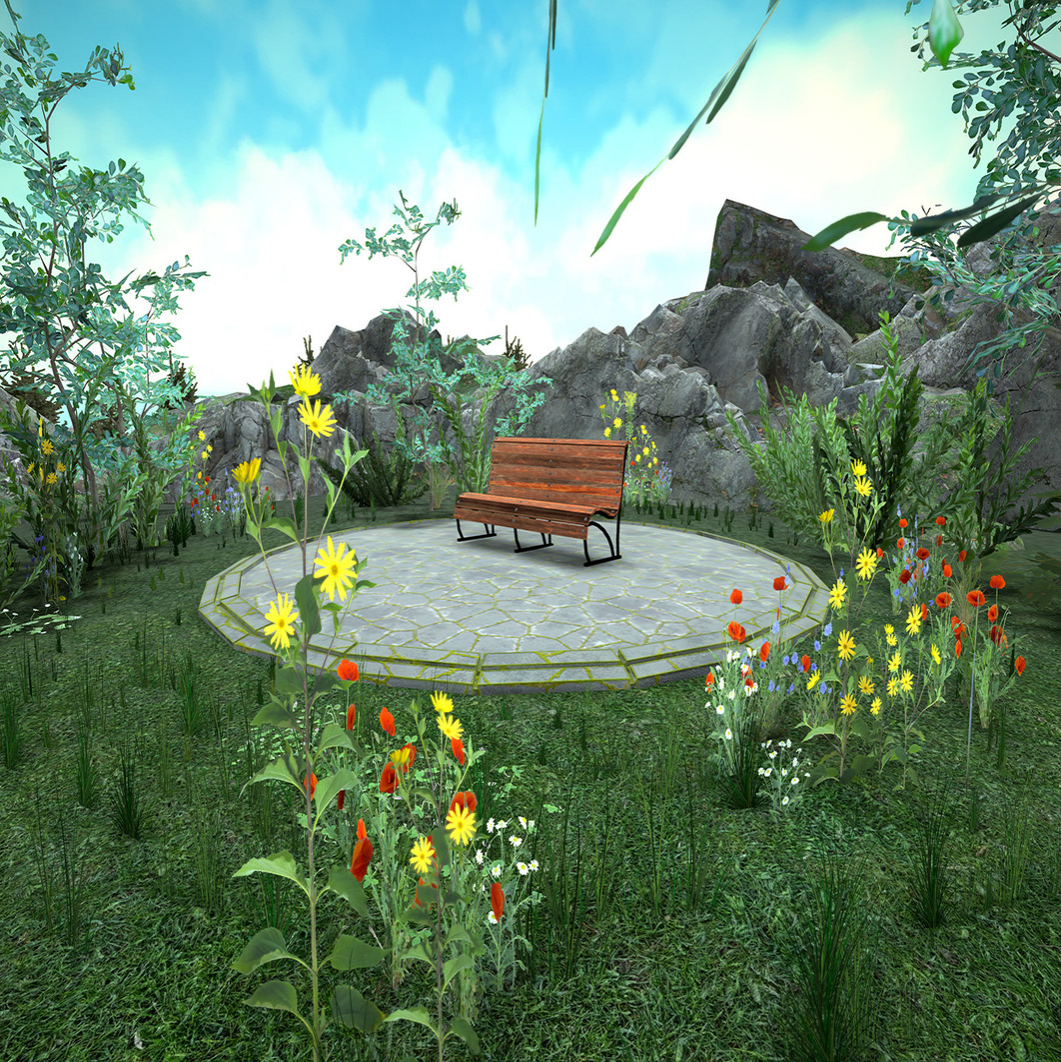
Screenshot of the virtual reality environment.

In the present study, the VR mindfulness intervention is delivered via a VR headset (Quest 2)^26^ and circumaural headphones (Bose Corporation)^27^ and was developed as a self-management intervention in the course of another project of our study group.^28^ Patients find themselves in a virtual natural environment, sitting on a park bench surrounded by flowers and trees overlooking a mountain lake (see figure 1). At the outset, patients are afforded a maximum of 5 min to acclimate themselves to the environment and then start the exercise independently. Under the guidance of a male narrator, patients are guided through an environment meditation consisting of a general opening sequence that suggests a brief recap of the day and includes elements of breathing meditation. In the main body of the meditation, the narrator directs the patient’s attention to different areas of the environment and asks the patient to describe them as accurately and as non-judgementally as possible. Special emphasis is placed on the description of colours and shapes. The final sequence contains short reflection questions on the content of the exercise. The exercise takes 15 min to complete.

The Quest 2 is a standalone VR system consisting of a mobile head-mounted display (HMD) with built-in stereo speakers and inside-out optical head motion tracking to provide 6 DoF positional tracking. The HMD is used with the flexible headstrap and weighs 503 g. Interaction with the VR environment can be performed using two separate hand controllers or integrated hand tracking. Patients were instructed to use the hand controllers. The Oculus Quest has two binocular OLED displays with a resolution of 1832×1920 pixels per eye, a refresh rate of 72 Hz and a field of view of ~100°. The VR intervention was created using Unity3D. Circumaural headphones are connected to the VR headset to avoid noise disturbance to other patients in the room.

The Quest 2 system setup involved the study team starting up the VR headset. This required configuring the guardian to the patient’s room and then selecting the study application from the main menu. The VR headset was then handed to the patient and adjusted to the shape of the patient’s head.

#### Music intervention

The receptive music intervention is delivered via an MP3-player (Apple iPod)^29^ and circumaural headphones (Bose Corporation).^27^ Prior to the first intervention, the participants can choose between four genres (classic, jazz, meditation and lounge) and will listen to this genre at both intervention time points. The playlists consist of music titles selected by a music psychologist according to the recommendations of Nilsson^30^ and used in a previous project of our group.^31^ They can be accessed in online supplemental appendix B. All tracks are instrumental and vary between 60 and 80 beats per minute (bpm). The music intervention lasts 15–20 min. Table 1 presents a comparative overview of the VR and the music intervention.

**Table 1:** Comparative overview of the virtual reality and the music intervention

Intervention design feature	VR intervention	Music intervention
Intervention type	Active	Receptive
Delivery format	Immersive VR environment via head-mounted display with headphones	Music via MP3 player and headphones
Technology	Quest 2 (Meta), Bose headphones	Apple iPod, Bose headphones
Intervention content	Mindfulness exercise, environment meditation in a natural environment with nature sounds	Listening to instrumental music (60–80 bpm)
Degree of user choice	Predefined meditation without choice options	Choice of four different music genres: classic, jazz, meditation or lounge
Type of guidance	Verbal guidance by a male narrator (directing attention to various aspects of the environment)	No guidance
Duration	15 min (+up to 5 min for acclimatisation)	15–20 min

bpm, beats per minute; VR, virtual reality.

#### Implementation and quality assurance of the intervention

To optimise intervention effectiveness, several strategies are being implemented. Recruitment data, reasons for non-participation (via a screening questionnaire) and dropout rates are systematically documented to assess feasibility. Following recruitment, participants receive standardised instructions for correct VR and music intervention application. Additionally, study staff are trained to provide consistent guidance and address technical difficulties.

To further ensure procedural consistency, a structured documentation form includes a checklist for standardisation and records start/end times and disruptions. Noise-cancelling headphones are used to minimise external distractions, ensuring participants can fully engage in the intervention.

Furthermore, weekly team meetings facilitate continuous process evaluation, allowing for structured feedback on data collection, recruitment challenges and potential implementation barriers. At the end of the study, feasibility and user experience data, including responses from an evaluation questionnaire, will be analysed to identify challenges and potential areas for improvement.

### Measures

Patients complete four different types of questionnaires during their study participation at the following measurement time points: after recruitment (T0; baseline), before the intervention (preintervention), after the intervention (postintervention) and at the end of the study (evaluation). All questionnaires were used in the German version. Additionally, physiological parameters (heart rate and blood pressure) are measured during the intervention phase. The preintervention, during-intervention and postintervention measurements are administered two times over two consecutive chemotherapy sessions (T1 and T2). Table 2 outlines the measures employed and the corresponding time points at which they are collected. Furthermore, saliva samples are collected both before and 25 min after the intervention, while heart rate and blood pressure are recorded at 5-minute intervals during the intervention. A detailed presentation of all measures used in the study is described in the following sections.

**Table 2:** Overview of measures used for the study and the measurement time points

Measure	Baseline	Preintervention	During intervention	Postintervention	Evaluation
Demographic data	X				
Cancer-related data	X				
VAS-social support	X				
Previous experiences with mindfulness and music for relaxation	X				
ASKU	X				
PHQ-4	X	X			
STAI-trait	X				
Salivary cortisol		X		X	
STAI-state		X		X	
PA-F-KF		X		X	
MIDOS-2		X		X	
VAS-anxiety		X		X	
VAS-relaxation		X		X	
Heart rate			X		
Blood pressure			X		
VAS-tolerability of chemotherapy				X	
VAS-perceived duration of chemotherapy				X	
PSSUQ*					X
VR-specific evaluation (self-developed)*					X
Music-specific evaluation (self-developed)†					X

* VR group only

† Music group only

ASKU, Short Scale for Measuring General Self-efficacy Beliefs; MIDOS-2, Minimal Documentation System version 2; PA-F-KF, Fear of Progression Questionnaire Short Form; PHQ-4, Patient Health Questionnaire-4; PSSUQ, Post-study System Usability Questionnaire; STAI, State-Trait Anxiety Inventory; VAS, Visual Analogue Scale; VR, virtual reality.

#### Baseline measures

##### Screening

In order to determine the reasons for a lack of interest in participating in the study, even though the patients addressed in the IAC fulfil the inclusion criteria, a screening form will be distributed. This screening form has been developed by the first authors on the basis of verbal feedback received during recruitment conversations on site. On this form, patients can tick items such as: ‘Concern about too much additional effort during therapy’, ‘No need, as chemotherapy is not perceived as unpleasant or stressful’ or ‘No interest in the research question or in the VR technology’. A refusal to provide further feedback is also possible, as well as a statement of unstated reasons. Completion of the screening questionnaire is voluntary and anonymous. The screening form can be viewed in online supplemental appendix C.

##### Generalized Anxiety Disorder-7 Questionnaire

Patients are screened for generalised anxiety using the German version of the GAD-7 questionnaire,^21^ translated by Löwe *et al*.^32^ It measures how much subjects have been bothered by the following problems over the past 2 weeks indicated on a 4-point Likert scale (0=‘not at all’, 1=‘for several days’, 2=‘more than half the days’, 3=‘nearly every day’): feeling nervous, anxious or on edge; not being able to stop or control worrying; worrying too much about different things; trouble relaxing; being so restless that it is hard to sit still; becoming easily annoyed or irritable; feeling afraid, as if something awful might happen. Participants can score a value between 0 and 21, with higher values indicating a higher degree of generalised anxiety symptoms. A recent study confirmed the reliability and validity of the GAD-7 in a German population sample with overall good internal consistency of α=0.85.^20^ Patients are screened for a score of at least 5, indicating mild generalised anxiety.^21^

##### Demographic and cancer-related data

Patients are asked to indicate their gender (female, male, diverse), age, highest educational level (based on the German educational system) and family status (unwed, in a partnership, married/civil partnership, divorced/civil partnership annulled, widowed/registered partner deceased).

Further, cancer-related data is assessed based on the Basic Documentation for Psycho-Oncology.^33 34^ Patients are asked to report their main cancer diagnosis, whether it is the first diagnosis and whether there has been a previous cycle of chemotherapy, what treatments they have received in the last 2 months, whether they have previously received psychiatric treatment or psychotherapy, and whether they are currently taking any psychopharmacological medication.

##### Short Scale for Measuring General Self-efficacy Beliefs

The Short Scale for Measuring General Self-efficacy Beliefs (ASKU)^35^ is a self-report based questionnaire assessing the participant’s perceived self-efficacy using three items on a 5-point Likert scale (1=‘never or very rarely true’; 5=‘very often or always true’). The ASKU reveals a reliability of between ω=0.81 and ω=0.86. The factorial validity can be described as sufficient with factor loadings of 0.77 and higher and the model fit is validated. The construct validity of the questionnaire was also validated by comparing the items to questionnaires assessing similar constructs.^35^

##### State-Trait Anxiety Inventory (STAI-trait)

The short trait-version of the State-Trait Anxiety Inventory (STAI)^36^ assesses trait-anxiety as a relatively stable personality characteristic and consists of 10 items that are rated on an 8-point Likert scale (1=‘not at all’; 8=‘completely’). Three of the 10 items have to be inverted prior to calculation. Total scores can range between 10 and 80, with higher scores indicating higher trait-anxiety. The trait-version also reveals excellent internal consistency ranging between α=0.88 and α=0.94 and the criterion validity was confirmed by correlating the items to other measures assessing similar constructs.^37^

### Primary outcome measure

#### State-Trait Anxiety Inventory (STAI-state)

The STAI^36^ is a self-report questionnaire that can assess both situational state-anxiety and stable trait-anxiety. The short version with 10 items was used to assess the situational state-anxiety as the primary outcome.^36^ The response for each of the 10 items is based on an 8-point Likert scale (1=‘not at all’; 8=‘completely’). Four items need to be inverted before calculating the total score. The STAI-state score ranges between 10 and 80. Higher values indicate higher state-anxiety. The STAI reveals excellent internal consistency of α=0.90.^36^

### Secondary outcome measures

#### Visual analogue scales

Visual analogue scales (VAS) are used to assess anxiety (‘How anxious do you feel in this moment?’ from ‘not anxious at all’ to ‘maximally anxious’), relaxation (‘How do you feel at this moment?’ from ‘maximally tense’ to ‘maximally relaxed’), social support (‘How much do you feel supported by your social network in the moment?’ from ‘not at all supported’ to ‘maximally supported’), tolerance of the chemotherapy session (‘How bearable did you perceive today’s chemotherapy treatment?’ from ‘minimally tolerable’ to ‘maximally tolerable’) and the perceived duration of the chemotherapy session (‘How quickly do you feel today’s chemotherapy session has gone so far?’ from ‘not quick at all’ to ‘maximally quick’). Patients are asked to rate their answer by marking a point on a 10 cm long line from 0 to 100, with 0 indicating total disagreement and 100 indicating total agreement. VAS are a quick and economical method for the assessment of different subjective states and symptoms.^38 39^

#### Patient Health Questionnaire-4

The German version of the Patient Health Questionnaire-4 (PHQ-4)^40^ assesses anxiety and depression using four items on a 4-point Likert scale (0=‘not at all’; 3=‘almost every day’). The values are added for the total score, higher values indicating a greater extent of anxiety and depression. The scale showed good internal consistency with α>0.80. A factor analysis revealed a good fit with 84% of the total variance explained. In a recent study, the internal consistency of the instrument for the German population could be again confirmed as good (ω=0.85).^41^

#### Fear of Progression Questionnaire Short Form

The Fear of Progression Questionnaire Short Form (PA-F-KF)^42^ consists of 12 items that are answered on a 5-point Likert scale (1=‘never’; 5=‘very often’). The questionnaire assesses the patient’s fear of progression on five dimensions (affective reaction, partnership/family, occupation, loss of autonomy) and shows overall good internal consistency (α=0.87).^42^ The construct validity of the items was confirmed by correlating the items to other measures assessing similar constructs (Hospital Anxiety and Depression Scale, HADS; Posttraumatic Stress Disorder Checklist - Civilian Version, PCL-C; Short Form-8 Health Survey, SF-8; Life Attitude Profile – Revised, LAP-R). A factor analysis revealed a one-dimensional structure, explaining 42% of the total variance.^42^ The psychometric properties were also confirmed in more recent studies. For example, a study by Hahn^43^ found an internal consistency of α=0.88 for the PA-F-KF.

#### Minimal Documentation System version 2

The Minimal Documentation System (MIDOS-2)^44^ is a self-report questionnaire assessing participants’ symptom-related afflictions due to chemotherapy-associated side effects on a 4-point Likert scale (0=‘none’; 3=‘major afflictions’). In total, there are 13 items. Ten items are measuring the side effects pain, nausea, regurgitation, shortness of breath, constipation, weakness, lack of appetite, fatigue, depression and anxiety. Two more items leave open spaces for naming other afflictions and one more item assesses how the participant is feeling that day, ranging from 0 (‘very bad’) to 4 (‘very good’). The sum score of each item describes the extent of complaints for each participant. The questionnaire reveals good psychometric properties, with internal consistencies varying between α=0.67 and 0.73 and the test-retest variability varying between r=0.69 and r=0.57.^44^ The psychometric properties were also confirmed for patient groups in non-palliative care.^45^

#### Heart rate and blood pressure

Heart rate and blood pressure are measured continuously from the beginning to the end of the intervention in 5-minute intervals in bpm using a pulse oximeter and blood pressure monitor. A member of the study team records these values while accompanying the intervention.

#### Saliva cortisol

Salivary samples are assessed to measure levels of cortisol in nmol/L. For this purpose, each participant insalivates a cotton swab for at least 30 s before the beginning of the intervention and 25 min after the end of the intervention. All saliva samples are stored in the dark at −20°C. Analyses will be carried out by Dresden LabService GmbH, Technical University of Dresden, Germany, using chemiluminescence immunoassay.

#### Evaluation

The evaluation was conducted for all three study arms, with tailored formats for each intervention group as well as the control group. For the VR group, seven items from the Post-Study System Usability Questionnaire^46^ (items 1, 6, 11, 16–19) were used. The full questionnaire consists of 21 items and was translated into German during a previous study conducted by our group.^47^ The selected items have been slightly adapted to better fit the specific VR application used in the study. Responses are given on a 7-point Likert scale (1=‘strongly disagree’; 7=‘strongly agree’), with the overall score reflecting the perceived usability of the VR system by the patients.

Additionally, patients were asked to rate several self-developed items that were tailored to the specific intervention (VR or music). These items assess whether the intervention was pleasing, relaxing, boring or annoying, whether it helped to alleviate any unpleasant feelings, induce calmness and create a pleasant atmosphere during the chemotherapy session. Patients also rated whether they liked the intervention, whether they would choose to use it again in a future chemotherapy session, whether they could focus more on the intervention than on their own thoughts, and whether they were already familiar with the intervention. All these items are also rated on a 7-point Likert scale (1=‘strongly disagree’ 7=‘strongly agree’).

Finally, all groups were asked to indicate which type of intervention (VR, music or none) they would prefer to use during a future chemotherapy session, rated on a 7-point Likert scale (1=‘not at all’; 7=‘very much’). The evaluation questionnaire also includes two additional free-text fields titled ‘Suggestions for enhancing the utilisation of VR/music’ and ‘Other comments’. The complete evaluation questionnaire is available for review in online supplemental appendix D.

### Recruitment and participant timeline

**Figure 2:**
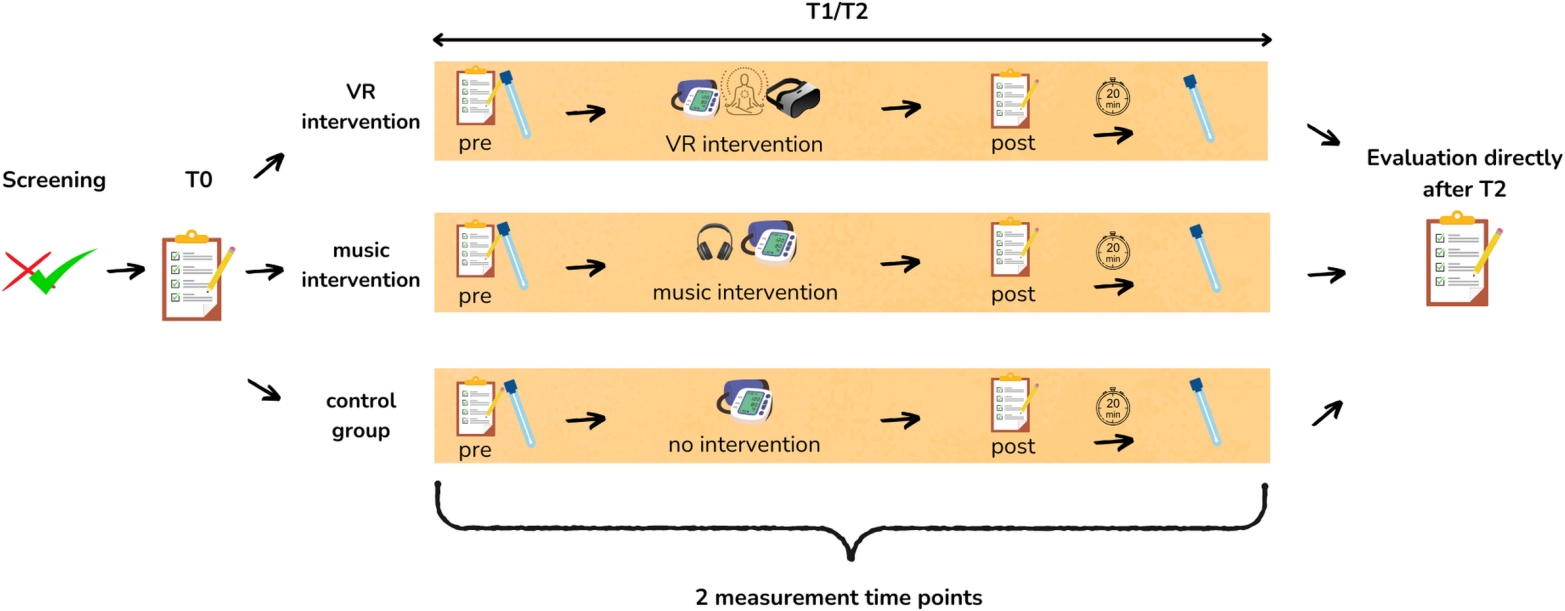
Participant timeline. VR, virtual reality.

The study team initiates contact with patients during their outpatient visit to the IAC and inquires about their interest in participating in the study. Subsequently, the treating physicians are consulted to confirm the patients’ eligibility for the study, and their approval is sought once eligibility is confirmed. Patients are then contacted again by the study team during their next chemotherapy session. All patients provide written informed consent prior to their participation in the study. Figure 2 shows the participant timeline. The patient information and consent form can be found in online supplemental appendix E.

The study team screens for general anxiety symptoms using the GAD-7 questionnaire.^20 21^ To ensure that participants are experiencing a level of anxiety significant enough to benefit from the interventions and demonstrate measurable changes in the primary outcome variable, only patients with at least moderate anxiety (defined as a score ≥5) are included in the study. Patients are randomly assigned to one of three intervention arms using computer-generated random numbers; however, they remain blinded to their group allocation until the first intervention time point (T1).

After recruitment, participants complete the first questionnaire for baseline assessment (T0). The intervention begins during the subsequent chemotherapy session at the IAC (T1). At this point, patients are informed of their study allocation and receive the preintervention questionnaire. Following the completion of the questionnaires, and after the flushing and premedication processes as part of the chemotherapy session, patients provide the first saliva sample by insalivating a cotton swab. The study team then assists patients in initiating the intervention.

Patients in intervention group 1 wear a VR headset and headphones and begin the mindfulness exercise. Patients in intervention group 2 put on headphones and listen to music. Patients in the control group 3 receive no additional treatment. A member of the study team remains with the patient, collecting data on heart rate and blood pressure at 5-minute intervals. After approximately 15–20 min, the intervention concludes. Patients then complete the postintervention questionnaire, and a second saliva sample is collected after 25 min. This procedure is repeated during the subsequent chemotherapy session (T2). Finally, patients in all groups receive a questionnaire specifically evaluating the intervention immediately after the second measurement time point. Participants in groups 2 and 3 are given the opportunity to try the VR intervention independently of data collection.

### Sample size

Based on previous literature, we estimate an effect size of 0.25. The G*Power V.3.1.9.7 software^48^ was employed to determine the requisite sample size. Assuming that the probability of error does not exceed α=0.05 and that the power (1−β) is at least 0.95, an analysis of variance with three groups and two time points with N=66 subjects can demonstrate an effect size of 0.25. Based on previous research projects, we calculate a drop-out rate of 20%, which increases the total sample size to N=82.

### Risk to patients

Possible risks that can arise, particularly during the VR intervention, are cybersickness^49^ and increased stress levels or anxiety due to the complex technical setup and instructions. In addition, the sensory isolation from the environment can lead to a feeling of loss of control. To counteract this, it was ensured that at least one person from the study team was always present with the patients during the intervention. Patients were able to contact the study team directly if they had any questions, problems or felt unwell. In addition, care was taken to ensure that patients were shielded from disturbances as much as possible. During the data collection phase, study personnel will be present to monitor and record any spontaneously occurring adverse events; if such events occur, they will be discussed with the treating physician and reviewed during the weekly team meetings. Patients will continue to receive ongoing medical care after the end of the trial, where potential side effects can still be discussed. In addition, short-term access to psycho-oncological support will be provided through the department of psychosomatic medicine and psychotherapy for patients who express a need for psychological support after study completion.

### Data monitoring and management

A randomisation file will be used to allocate a participant code to the intervention arms. A recruitment file is used to allocate the participant code to all included patients and, further, contains patients’ diagnoses, medical clearance and information about the recruitment process (treating physicians’ approval for study participation, checklist for how many attempts were needed to meet the patient in the IAC, date of informed consent). A data file contains pseudonymised survey data of patients for each measurement time point. Another file will be used to anonymously assess drop-outs, screening failures and reasons for non-participation or lack of interest. The data will be entered by the study team accountable for implementation of the study and will be double-checked by another team member. Supervision by the study team during chemotherapy sessions ensures the questionnaires are fully completed by participants. Data records will be kept separate from the consent forms. Data records of patients who did not meet the inclusion criteria (screening failures) will be disposed of in accordance with data protection regulations. After collection, salivary cortisol samples will be temporarily stored in a freezer (−20°C) and sent to a laboratory (Dresden LabService GmbH, Technical University of Dresden, Germany). Questionnaires and cortisol levels derived from saliva samples will be stored as combined research data for at least 10 years (in accordance with the principles of Good Clinical Practice). Access to the anonymised final trial dataset will be granted on request to the study team; the intended use of the data must be disclosed, after which the appropriate dataset will be provided.

Patients will receive standard medical care during the whole participation. No adverse side effects are expected due to the interventions and participating patients do not face higher risks compared with patients who are not participating in the current study. As a member of the study team will supervise each session, participants can terminate the intervention at any time. Participants can report any side effects to the study team and use the evaluation questionnaires for thorough feedback. Therefore, study monitoring is not needed.

### Randomisation, blinding and treatment allocation

Randomisation is conducted using random numbers. Using a computer program (Microsoft Excel, Microsoft Corporation), a team member who does not participate in recruiting patients generates an Excel sheet with numbers representing the three intervention groups (using the command ‘=RANDBETWEEN(1,3)’). The random allocation sequence assigns patients in equal proportions to one of the three intervention groups. In this sheet, the numbers are masked until a new patient is enrolled, which assures that members of the study team accountable for participant recruitment are blinded until patients’ consent. Patients are blinded about their study arm allocation until the first intervention time-point (T1). There is no further blinding of the study team.

### Statistical analysis

All characteristics collected in the study are described in detail using descriptive methods. Qualitative characteristics are indicated by absolute and relative frequencies. Quantitative characteristics are described using mean values, SD, median, minimum and maximum. Parametric analysis methods are used to evaluate the primary and secondary target variables. If assumptions for parametric tests are violated, appropriate non-parametric alternatives will be considered.

Situational anxiety as the primary outcome (measured using the STAI-state) is analysed using separate 3×2 mixed factorial analyses of variance (ANOVAs) for each of the two chemotherapy sessions, with the between-subject factor ‘intervention’ (VR vs music vs control) and the within-subject factors ‘time’ (before vs after intervention). A probability of error of α=0.05 is set. The unit of analysis is individual patients. Effect sizes will be reported using partial eta squared (η²) for ANOVA analyses. Cohen’s d is calculated for post-hoc comparisons when appropriate. The secondary outcome criteria are analysed exploratively, also using ANOVAs and post hoc tests as appropriate. There is no adjustment of the probability of error. The statistical analysis is carried out with the help of standard statistical software (SPSS V.29). Participants will be included in the statistical analysis if they have completed both assessment time points and received the intervention for a minimum of 10 min per session. Furthermore, the intervention must be administered in consecutive sessions as scheduled. Participants who do not meet these criteria will be classified as dropouts. Both an intention-to-treat analysis and a per-protocol analysis will be performed. The results will be compared and discussed.

The feasibility of the intervention will be evaluated based on multiple data sources. A descriptive analysis of the screening and recruitment data will provide insights into patient participation and reasons for non-participation. Additionally, feasibility will be assessed based on study staff documentation during data collection sessions, as well as recorded reasons for dropout, and potential side effects. Furthermore, the evaluation questionnaire, which includes both usability assessment and self-developed items, will be analysed. The participants’ comments in the free text fields of the evaluation questionnaire will be categorised based on their content into issues and suggestions for improvement.

### Protocol amendments

Protocol amendments were requested initially on 4 November 2022 to extend the sample due to a lack of subjects matching the inclusion criteria. Before the amendment, participants consisted solely of patients with a gynaecological or senological diagnosis. After the amendment, participants with gastrointestinal and haematological diagnoses were also included. To improve the homogeneity of the sample, the GAD-7 with its cut-off criterion of a value ≥5 was introduced as a screening tool. These protocol amendments were granted on 19 November 2022.

A second request to amend the protocol was issued on 3 August 2023 to introduce a self-constructed screening questionnaire for further inquiry into reasons why patients may be disinterested in participation in the study. This protocol amendment was granted on 9 August 2023. All amendments are reflected in protocol version #4, dated 17 March 2025.

### Ethics and dissemination

The ethics committee of the medical faculty of Heinrich-Heine-University Düsseldorf has been obtained (2022-1880). Divergences or modifications of the study protocol will be documented and relevant cases will be reported to the ethics committee. The trial is registered at the German Clinical Trials Register (DRKS00029738), dated 16 August 2022. For all processed data adherence to data protection regulations is warranted. Written informed consent is obtained from patients by the study team and patients are informed that they can withdraw their consent without explanation at any time without facing any consequences. Further, patients are informed that all stored personal data can be deleted on demand at any point. All personal identifiers will be pseudonymised. The final data set can be assessed only by the study team. After the end of the study, the ethics committee will be informed within 90 days.

Findings of the trial will be reported by publishing in international scientific, peer-reviewed journals. Given several outcome parameters, several publications are planned. Further, presentations at conferences are planned. Until publication, the study team commits to full disclosure of the trial results.

### Patient and public involvement

Patients and the public were not involved in the design, conduct or reporting of this study. The research questions, study design, outcome measures, recruitment strategies and dissemination plans were developed by the research team without direct input from patients, carers or members of the public. This decision was made based on the specific nature and feasibility constraints of the study.

## Supplementary material

10.1136/bmjopen-2024-094040 online supplemental file 1

10.1136/bmjopen-2024-094040 online supplemental file 2

10.1136/bmjopen-2024-094040 online supplemental file 3

10.1136/bmjopen-2024-094040 online supplemental file 4

10.1136/bmjopen-2024-094040 online supplemental file 5
