# Supplementary material for: Virtual Reality and Sound Intervention under Chemotherapy (ViSu): study protocol for a three-arm randomised-controlled trial
Source: BMJ Open. 2025 Apr 9;15(4):e094040. doi: 10.1136/bmjopen-2024-094040 (PMC11987127; doi:10.1136/bmjopen-2024-094040)
Supplement: online supplemental file 2 [file bmjopen-15-4-s002.docx]

Appendix B

Music Title Overview

|  |  | **Duration** |
| --- | --- | --- |
| **Jazz** | 1. Bill Evans, Danny Boy 2. The Niall O’Sullivan Quartet, Moon Love 3. Keith Jarrett, Don’t ever leave me 4. Billy Higgins, Silence 5. Keith Jarrett, Over the Rainbow 6. Keith Jarrett, Paint my Heart Red 7. Bill Evans, Some Other Time 8. Miles Davies, My funny Valentine 9. Bill Evans, When I fall in Love 10. Coltrane Quartet, It’s easy to remember 11. Billy Higgins, Round Midnight | 3:41  4:16  3:22  8:45  6:02  6:15  5:19  6:02  4:54  2:47  11:36 |
| **Klassik** | 1. Johann Sebastian Bach – Orchestersuite Nr. 3 D-Dur („Air“) 2. Yiruma – River Flows In You 3. Claude Debussy – Prelude No. 8 „La Fille aux cheveux de lin“ 4. Wolfgang Amadeus Mozart – Serenade in G, K. 525 „Eine Kleine Nachtmusik“ 5. Camille Saint-Saëns – Le carnaval des animaux – le cygne 6. Frédéric Chopin - Nocturne in Es-Dur, Op. 9, No. 2 7. Georg Friedrich Händel – Wassermusik, Suite Nr. 1 in F-Dur, „Air“ 8. Gabriel Fauré – Pavane, Op. 50 9. Claude Debussy – Suite bergamasque „Clair de lune“ 10. Wolfgang Amadeus Mozart – Violinkonzert No.3, 2. Satz: Adagio 11. Wolfgang Amadeus Mozart – 21. Klavierkonzert, 2. Satz: Andante 12. Edvard Grieg – Peer-Gynt-Suite Nr. 1, „Morgenstimmung“ 13. Wolfgang Amadeus Mozart – Klarinettenkonzert, 2. Satz: Adagio 14. Ludovico Einaudi – Le Onde 15. Georg Friedrich Händel – Oboenkonzert Nr. 1, 1. Satz: Adagio 16. Philip Glass – Metamorphosis Two | 5:28  3:06  2:47  3:35  2:50  4:05  7:36  5:37  4:39  8:49  7:07  3:53  7:00  5:24  3:26  7:17 |
| **Lounge** | 1. Chillout 2. Wonderful Chill Out Music 3. Portrait of Me 4. Sex Music 5. Brand New (Inspirational Music) 6. Life 7. Real and True 8. Weekend Lounge 9. Pilates Music 10. Meditation Music for Relaxation 11. Soft Chillout Music 12. Stop (Breathing and Positive Thou) 13. Chilled Moods 14. Pure Chill Out | 6:10  4:55  5:08  5:01  6:10  4:45  4:39  5:54  5:08  4:48  3:54  4:42  5:28  6:22 |
| **Meditation** | *CD: MusiCure 1. The Journey*   1. Titel 1 2. Titel 2 3. Titel 3 4. Titel 4 5. Titel 5 6. Titel 6 7. Titel 7 | 15:37  15:23  9:30  11:49  9:20  3:11  5:53 |
