# Supplementary material for: Virtual Reality and Sound Intervention under Chemotherapy (ViSu): study protocol for a three-arm randomised-controlled trial
Source: BMJ Open. 2025 Apr 9;15(4):e094040. doi: 10.1136/bmjopen-2024-094040 (PMC11987127; doi:10.1136/bmjopen-2024-094040)
Supplement: online supplemental file 3 [file bmjopen-15-4-s003.docx]

| 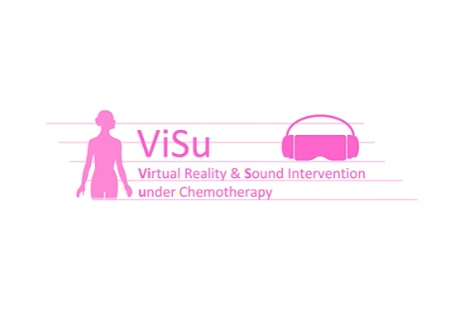  **Screening Form** | Patient-ID:  (only after inclusion) |  |
| --- | --- | --- |

| Date of Screening: | \|  \|  \| **.** \|  \|  \| **.** \| **2** \| **0** \| **2** \|  \| \| --- \| --- \| --- \| --- \| --- \| --- \| --- \| --- \| --- \| --- \| |
| --- | --- | --- | --- | --- | --- | --- | --- | --- | --- | --- | --- |

**Inclusion Criteria**

| Age of patient: | ≥18 years  <18 years |
| --- | --- |
| Does the patient have sufficient language skills to participate? | yes  no |
| Is there a confirmed diagnosis of cancer? | yes  no |
| Is the patient receiving intravenous chemotherapy (duration of application  including flushing at least 60 minutes)? | yes  no |
| Will the first survey take place at least at the second chemotherapy session? | yes  no |
| At least five chemotherapy sessions outstanding? | yes  no |
| **GAD-7 ≥ 5**? | yes  no |

**Exclusion Criteria**

| Are there any serious visual and/or hearing impairments? | yes  no |
| --- | --- |
| Are there any relevant pre-existing conditions?^[[1]](#footnote-1)^ | yes  no |
| Were there any serious side effects from the first chemotherapy? | yes  no |
| Is the chemotherapy being carried out as part of another study? | yes  no |
| Are there any brain metastases? | yes  no |

**Inclusion**

| Education has taken place | yes  no |
| --- | --- |
| Inclusion and exclusion criteria enable participation | yes  no |

| Does the patient consent to participate in the study? | yes  no |
| --- | --- |

In case of “Yes“, the declaration of consent will be signed. In case of “No”, the second page will be filled in.

| 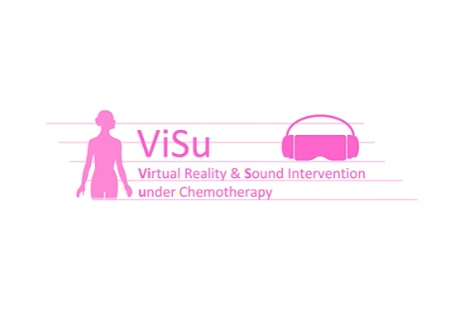 | **Screening Form** |
| --- | --- |

Please check all that apply:

*Multiple responses possible*

Own current state of health (physical, mental) speaks against participation.

Focus should be on chemotherapy, participation is perceived as disruptive.

Concern about too much additional effort during therapy.

Concern about possible side effects.

Concern about data protection.

Concern about the collection of saliva samples.

No need, as chemotherapy is not perceived as unpleasant or stressful.

No need, as own strategies for distraction and bridging time are used (reading, smartphone/tablet, accompanying person, fellow patients, sleeping,…)

No interest in participating in clinical trials in general.

No interest in the question or in the VR technology.

No interest in or skepticism about the effectiveness of the interventions.

No interest in psycho-oncological support services.

If none of the points above apply or if further comments should be made, the free text field below may be used:

|  |
| --- |

No further indications of reasons.

**Many thanks!**

_________________________________________________________________

Patient did not want to provide feedback. *(Only to be filled in by the research team)*

1. Neurological/psychiatric pre-existing conditions that affect the vestibular system, impair the sense of balance or alter visual perception, epilepsy or claustrophobia. [↑](#footnote-ref-1)
