# Supplementary material for: Virtual Reality and Sound Intervention under Chemotherapy (ViSu): study protocol for a three-arm randomised-controlled trial
Source: BMJ Open. 2025 Apr 9;15(4):e094040. doi: 10.1136/bmjopen-2024-094040 (PMC11987127; doi:10.1136/bmjopen-2024-094040)
Supplement: online supplemental file 4 [file bmjopen-15-4-s004.docx]

| 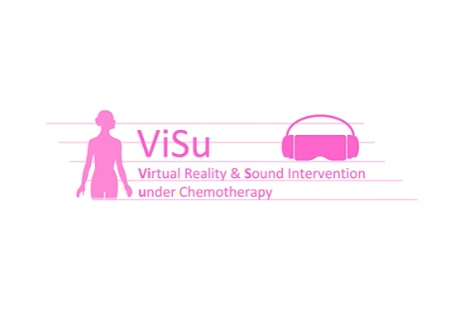 | Date: |  |
| --- | --- | --- |
| **Evaluation Form VR Group** | Patient-ID: |  |

Below you are asked to rate the user-friendliness of the virtual reality application you have just tested. Please read each statement and mark the answer option that best applies to you.

|  | does not apply at all | | | | applies completely | | | |  |
| --- | --- | --- | --- | --- | --- | --- | --- | --- | --- |
|  | 1 | 2 | 3 | 4 | | 5 | 6 | 7 | |
| Overall, I am pleased with how easy it was to use the VR application. | □ | □ | □ | □ | | □ | □ | □ | |
| I felt comfortable while using the VR application. | □ | □ | □ | □ | | □ | □ | □ | |
| The information presented through the VR application was clearly understandable. | □ | □ | □ | □ | | □ | □ | □ | |
| The presentation and design of the VR mindfulness exercise was pleasant. | □ | □ | □ | □ | | □ | □ | □ | |
| I enjoyed the VR application. | □ | □ | □ | □ | | □ | □ | □ | |
| The application of virtual reality offers all the possibilities I expected from it. | □ | □ | □ | □ | | □ | □ | □ | |
| Overall, I am satisfied with the use of the VR application. | □ | □ | □ | □ | | □ | □ | □ | |
| Wearing the VR glasses was comfortable. | □ | □ | □ | □ | | □ | □ | □ | |
| The VR glasses were disruptive. | □ | □ | □ | □ | | □ | □ | □ | |
| The VR mindfulness exercise was pleasant. | □ | □ | □ | □ | | □ | □ | □ | |
| The VR mindfulness exercise was relaxing. | □ | □ | □ | □ | | □ | □ | □ | |
| I would like to do this or another VR mindfulness exercise again at a future appointment. | □ | □ | □ | □ | | □ | □ | □ | |
| The VR mindfulness exercise helped me to tolerate unpleasant feelings. | □ | □ | □ | □ | | □ | □ | □ | |
| The VR mindfulness exercise made the atmosphere pleasant during chemotherapy. | □ | □ | □ | □ | | □ | □ | □ | |
| The VR mindfulness exercise calmed me down. | □ | □ | □ | □ | | □ | □ | □ | |
| I enjoyed the VR mindfulness exercise. | □ | □ | □ | □ | | □ | □ | □ | |
| I already knew the mindfulness exercise. | □ | □ | □ | □ | | □ | □ | □ | |

Three interventions were compared in this project: the use of a VR mindfulness exercise, the use of music (selection from four genres: meditation, classical, lounge, jazz) and a control condition without intervention.

**Please rate the statements by ticking the box that most closely matches your feelings:**

|  | Not at all | | | | Very much | | | |
| --- | --- | --- | --- | --- | --- | --- | --- | --- |
|  |  | | | | | | | |
| At my next chemotherapy appointment, I would like to use the VR application again. | □ | □ | □ | □ | | □ | □ | □ |
| At my next chemotherapy session, I would like to listen to music (selection from the genres mentioned above). | □ | □ | □ | □ | | □ | □ | □ |
| At my next chemotherapy session, I would like to use neither the VR mindfulness exercise nor the music (selection from the genres mentioned above). | □ | □ | □ | □ | | □ | □ | □ |

Do you have any suggestions for improving the use of VR glasses?

|  |
| --- |

Other notes:

|  |
| --- |

Thank you for your participation!

| 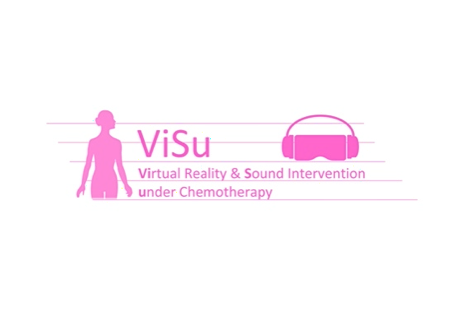 | Date: |  |
| --- | --- | --- |
| **Evaluation Form Music Group** | Patient-ID: |  |

Please check the box that best reflects your feelings.

|  | does not apply at all | | | | | | | applies completely | | | | | | |
| --- | --- | --- | --- | --- | --- | --- | --- | --- | --- | --- | --- | --- | --- | --- |
|  | 1 | 2 | | 3 | | 4 | | | 5 | | 6 | | 7 | |
| The music intervention was pleasant. | □ | | □ | | □ | | □ | | | □ | | □ | | □ |
| The music intervention was relaxing. | □ | | □ | | □ | | □ | | | □ | | □ | | □ |
| I would like to hear this or similar music again at my next appointment. | □ | | □ | | □ | | □ | | | □ | | □ | | □ |
| The music was disruptive. | □ | | □ | | □ | | □ | | | □ | | □ | | □ |
| The music helped me to endure unpleasant feelings. | □ | | □ | | □ | | □ | | | □ | | □ | | □ |
| The music made the atmosphere pleasant during chemotherapy. | □ | | □ | | □ | | □ | | | □ | | □ | | □ |
| The music calmed me down. | □ | | □ | | □ | | □ | | | □ | | □ | | □ |
| I enjoyed the music. | □ | | □ | | □ | | □ | | | □ | | □ | | □ |
| I already knew some of the songs. | □ | | □ | | □ | | □ | | | □ | | □ | | □ |

Three interventions were compared in this project, the use of a VR mindfulness exercise, the use of music (selection from four genres: meditation, classical, lounge, jazz) and a control condition without intervention.

**Please rate the statements by ticking the box that most closely matches your feelings:**

|  | Not at all | | | | Very much | | | |
| --- | --- | --- | --- | --- | --- | --- | --- | --- |
|  |  | | | | | | | |
| At my next chemotherapy appointment, I would like to listen to music again (selection from the genres mentioned above). | □ | □ | □ | □ | | □ | □ | □ |
| At my next chemotherapy appointment, I would like to use the VR application. | □ | □ | □ | □ | | □ | □ | □ |
| At my next chemotherapy appointment, I would like to use neither the VR mindfulness exercise nor the music (selection from the genres mentioned above). | □ | □ | □ | □ | | □ | □ | □ |

Do you have any suggestions for improving the use of music?

|  |
| --- |

Other notes:

|  |
| --- |

Thank you four your participation!

| 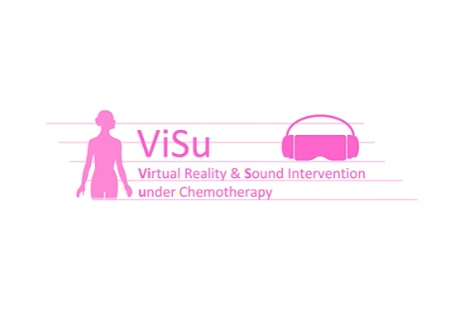 | Date: |  |
| --- | --- | --- |
| **Evaluation Form Control Group** | Patient-ID: |  |

In this project, three interventions were compared: the use of a VR mindfulness exercise, the use of music (selection from 4 genres: meditation, classical, lounge, jazz) and a control condition without intervention. **Please rate the statements by checking the box that most closely matches your feelings:**

|  | Not at all | | |  | |  | |  | Very much | |
| --- | --- | --- | --- | --- | --- | --- | --- | --- | --- | --- |
|  |  | | | | | | | | | |
| At my next chemotherapy appointment, I would like to use the VR application. | □ | □ | □ | | □ | | □ | | □ | □ |
| At my next chemotherapy appointment, I would like to listen to music (selection from the genres mentioned above). | □ | □ | □ | | □ | | □ | | □ | □ |
| At my next chemotherapy appointment, I would like to use neither the VR mindfulness exercise nor the music (selection from the genres mentioned above). | □ | □ | □ | | □ | | □ | | □ | □ |

Do you have any suggestions for improving the use of music?

|  |
| --- |

Other notes:

|  |
| --- |

Thank you for your participation!
