# Supplementary material for: Virtual Reality and Sound Intervention under Chemotherapy (ViSu): study protocol for a three-arm randomised-controlled trial
Source: BMJ Open. 2025 Apr 9;15(4):e094040. doi: 10.1136/bmjopen-2024-094040 (PMC11987127; doi:10.1136/bmjopen-2024-094040)
Supplement: online supplemental file 5 [file bmjopen-15-4-s005.docx]

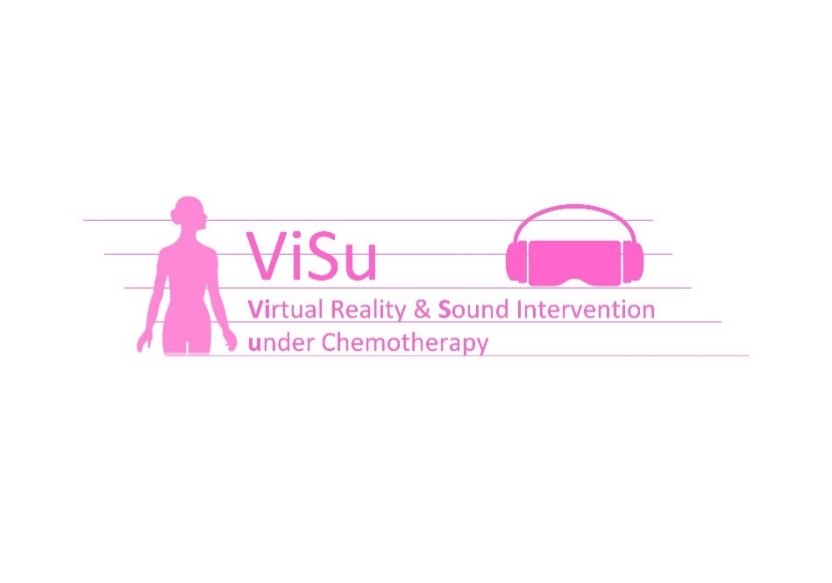
**Patient information on participation in the ViSu-study**

Dear patient,

You are invited to take part in a clinical trial. A clinical trial is conducted for research purposes, for example to gain further insights into a known treatment method. In the following, we would like to inform you about the objectives and the course of the study and explain why your cooperation is important if you take part in the study.

Participation in this study is voluntary. If you participate in the study, you have the right to terminate the study prematurely at any time without giving reasons. Should you decide not to participate or to terminate the study prematurely, this will not lead to any disadvantageous treatment or a worse treatment outcome. We ask you to read this information carefully and then decide whether or not you wish to participate in this study.

**Study title:** ViSu-study: Virtual Reality & Sound Intervention under Chemotherapy

**Client and study management:**

| Dr. med. André Karger (MME)  Clinical Institute für Psychosomatic Medicine and Psychotherapy  University Hospital Düsseldorf, A. ö. R.  Moorenstr. 5  40225 Düsseldorf  Tel. 0211/8116221 |  | PD Dr. Nora K. Schaal  Institute for Experimenal Psychology  Heinrich-Heine-University Düsseldorf  Universitätsstraße 1  40225 Düsseldorf  Tel. 0211/8113863 |
| --- | --- | --- |

**What is the study about?**

Music interventions and virtual reality (VR) technology have already demonstrated their anxiety-relieving effect during various medical treatments in different studies. Both techniques may be suitable methods for alleviating anxiety and stress during chemotherapy. The present experimental, clinical study aims to investigate the effect of VR and music intervention during chemotherapy on situational anxiety and situational stress. For this purpose, gynecologic and senologic oncology patients who are at least in the second cycle of chemotherapy are included in the study and randomly assigned to one of the three study arms: (1) active intervention with VR mindfulness exercise, (2) receptive music intervention, or (3) control group with standard care.

Participation in this study is voluntary. You will only be included if you give your written consent. If you do not wish to participate in the study or wish to withdraw from it at a later date, you will not suffer any disadvantages as a result. You can withdraw your consent verbally or in writing at any time, even without giving reasons.

The study was submitted to the responsible ethics committee. It has raised no objections.

**How does the study work?**

During your consultation at the Breast Center or the gynecological outpatient clinic of the University Women's Hospital of the UKD, your attending physician will inform you about the study and ask you about your general interest. Your interest was communicated to the investigator or a member of the study team, who then visited you for your first chemotherapy cycle at the Interdisciplinary Outpatient Chemotherapy Center (IAC) to enroll you in the study. If you wish to participate in the study, we will first ask you for your written consent after you have been informed about the study verbally by the investigator or a member of the study team and have received this written patient information. Once you have signed the consent form, you will be assigned to one of the three groups. This happens randomly and nobody can influence it.

The study will start from the second cycle of your chemotherapy at the earliest. On this day, you will be visited by a member of the study team and, if you have been assigned to group (1) or (2), you will be familiarized with the VR or music intervention technique. This will take place during the premedication of the cycle. In addition to the technique, but irrespective of your group affiliation, you will also be fitted with a pulse oximeter, which will record your pulse during the entire examination. Before the administration of the cytostatic drugs starts, you will receive the first questionnaire from a member of the study team, which you will fill out before the start. As soon as the administration of cytostatic drugs starts, the member of the study team will take your first saliva sample. Your intervention (group (1) and (2)) or a period of approximately 20 minutes will start immediately afterwards. The following will happen during each intervention:

1. VR group: the member of the study team will put the VR goggles with the corresponding headphones on you. Then the intervention will start, an ambient meditation that you will perform in a daytime environment. The exercise is accompanied by a male speaker and is accompanied by ambient nature sounds. The ambient meditation lasts around 20 minutes.
2. Music group: The member of the study team will put the headphones on you and play the music you have chosen beforehand. You have a choice of four different playlists, divided by genre: Classical, Lounge, Meditation and Jazz. Each playlist covers a period of at least 30 minutes. After about 20 minutes, the member of the study team will stop the music and end the intervention.
3. Control group: They will receive the usual standard care of the IAC.

Before the administration of cytostatic drugs is finished, i.e. after about 20 minutes, the member of the study team will inform you that the intervention is over and take the technical equipment ((1) glasses and headphones, (2) headphones) back from you. The second saliva sample will then be taken. You will then receive the second questionnaire, which you will complete immediately.

The study design provides for two surveys, i.e. after this first survey on the second chemotherapy cycle at the earliest, there will be another survey on the following cycle. You will also be seen by a member of the study team on the same day for this chemotherapy cycle and will receive the same intervention for this appointment as for the first survey.

A member of the study team will be close to you at all times during the first and second appointments. The member will ensure that the cytostatic drugs are administered without any problems and will also be able to contact the medical staff. In this way, you can concentrate on the intervention.

**What are the benefits and risks of your participation?**

**Benefit:** By participating in the study, you will not gain any further benefit in this situation apart from the presumed benefit of the VR or music intervention. Likewise, due to the randomized design of the study, neither you nor your doctor can influence which arm of the study you are drawn into. No financial compensation is provided. The results of this study may help to improve care for other patients suffering from your disease.

**Risks:** Participation in the study is not expected to have any adverse consequences for you. The planned saliva samples are harmless and are only used to determine the cortisol level in your saliva. However, we would like to point out that saliva may also contain genetic information; however, no genetic analyses will be carried out as part of the study.

**What data will be collected?**

The following research data will be collected from you as part of the study:

- During the entire survey, your pulse will be recorded using a pulse oximeter.
- Saliva samples are collected as part of the scientific investigation of the body's own hormone cortisol, which is generally released in humans, e.g. in stress reactions. The examination of saliva is used exclusively to determine cortisol levels and for basic research. No genetic studies are carried out with it. The saliva samples are collected using a cotton swab, which should be salivated for approx. 30 seconds. You are not expected to derive any direct benefit from such tests. Your consent to the use of the collected saliva for research purposes is, of course, voluntary. You can withdraw your consent at any time without giving reasons. Of course, this will not result in any disadvantages for you. If you withdraw your consent, your stored saliva sample will be destroyed immediately.
- In the questionnaire, you will be presented with established questionnaire instruments that are obvious to you. These relate to your situational anxiety, your current physical well-being, your cancer-related fears, your satisfaction with chemotherapy and your self-efficacy expectations.
- Socio-demographic data (age), information about your illness and treatment

**What happens to my data?**

Data storage is pseudonymized. Pseudonymization is the replacement of the name and other identifying features with an identifier for the purpose of excluding or significantly complicating the identification of the data subject (according to Art. 4 para. 5 GDPR). If you are included in the study, you will receive a participant ID consisting of an alphanumeric character string that has no reference to your person. All research data collected from you (saliva samples, pulse, questionnaires) will be identified solely by this participant ID and linked to each other and merged into a pseudonymized data table. In addition, the participant ID allows us to assign your contact data, which we store in another password-protected list separately from all other data collected during your surveys on a secure internal NAS server of the Clinical Institute for Psychosomatic Medicine and Psychotherapy of the University Hospital Düsseldorf, and is used in the event of corrections or revocation of data by you as a participant. The NAS server is a secure server of the UKD, which offers an increased security standard. Only an employee who is bound to secrecy and confidentiality and trained in data protection has access to this list and is not involved in the processing of your research data.

The information from the questionnaire and the evaluated saliva samples are manually transferred to the superordinate, pseudonymized data table by employees who are bound to secrecy and confidentiality and have been trained in data protection and who do not have access to the allocation list. The questionnaires are stored in locked cabinets in folders in lockable rooms of the Clinical Institute for Psychosomatic Medicine and Psychotherapy of the University Hospital Düsseldorf, to which only employees of the Clinical Institute for Psychosomatic Medicine and Psychotherapy have access.

After completion of the study, the assignment list will be deleted and thus an assignment to the collected research data is no longer possible. The data collected is subject to the currently valid data protection legislation. The legal basis for the processing of the aforementioned personal data is the declaration of consent in accordance with Art. 6 (1) letter a and Art. 9 (2) letter a EU GDPR. All data collected as part of the study will be stored for a period of 10 years in accordance with the guidelines of good scientific practice of the German Research Foundation and the model professional code of conduct for physicians and will be completely destroyed at the end of this period. This does not include your contact details, which we may use to contact you for the purpose of future studies, provided you have consented to this. Deletion will then take place in the event of revocation or if contact cannot be established permanently. Deletion at the subject's request is excluded from this. The anonymized data will be evaluated after completion of the study. A biometric report is created.

**Measures to protect your data**

We have taken various organizational and technical measures to protect your data. The organizational measures include data protection training for all employees involved in this study as well as access and key authorization for the designated storage locations. In addition, the computers on which the electronic data is stored are password-protected and only connected to the local hospital network. The electronic data storage locations are also access-controlled and password-protected. The allocation list is protected with an additional password, stored separately from the rest of the survey data and can only be accessed by an employee who is bound to secrecy and confidentiality and trained in data protection and who is not involved in the evaluation of the higher-level data table.

**Consent to the use of your address for future projects**

The Clinical Institute for Psychosomatic Medicine and Psychotherapy at Düsseldorf University Hospital and the Institute for Experimental Psychology at Heinrich Heine University Düsseldorf would like to continue their research in the future. We would therefore like to process your address in order to inform you about similar research projects in the future. You are of course free to decide whether you actually take part in one of these projects.

**Scientific and commercial use of the research results**

The results of this research project may be published in scientific journals and at conferences. These publications will not contain any personal data that could be used to identify you personally. It is also possible that the research results may be used commercially, e.g. patented. You will not participate in any possible commercial benefit.

**Information on data protection in accordance with Art. 13 GDPR**

*Data controller*

The controller within the meaning of Art. 4 No. 7 EU General Data Protection Regulation (GDPR) is André Karger, Clinical Institute for Psychosomatic Medicine and Psychotherapy, University Hospital Düsseldorf (UKD), A ö. R., Moorenstraße 5 in 40225 Düsseldorf, 0211/81-16212, and Nora Schaal, Institute for Experimental Psychology, Heinrich Heine University (HHU) Düsseldorf, 0211/81-13863. If you have any concerns, questions or complaints regarding data processing and compliance with data protection requirements, please contact the *study management* first (*see above for contact details*). For further questions or in case of problems regarding data protection, please contact the data protection officer of the UKD and the HHU at

| Data Protection Officer UKD  Data Protection Office  Moorenstraße 5  40225 Düsseldorf  Phone: 0211 81-00  datenschutz@med.uni-duesseldorf.de | Data Protection Officer HHU  Data Protection Office  Universitätsstraße 1  40225 Düsseldorf  Phone: 0211 81-13060  datenschutz@uni-duesseldorf.de |
| --- | --- |

*Purposes of processing and legal basis*

Your personal data will be processed exclusively for the purposes stated in the declaration of information and consent. The legal basis for the processing is your voluntary and express consent within the meaning of Art. 6 para. 1 sentence 1 lit. a, 9 para. 2 lit. a GDPR. You have the right to withdraw this consent at any time with effect for the future. This does not affect the lawfulness of the data processing carried out up to the point of withdrawal.

*Deletion periods*

If you withdraw your consent, your personal data will be deleted by the UKD and HHU in accordance with data protection regulations, provided that there are no statutory retention obligations to prevent immediate deletion.

*Your rights*

1. the right to information, including a free copy, as to whether and which of your data is being processed (Art. 15 EU GDPR)
2. the right to request the rectification or completion of data concerning you (Art. 16 EU GDPR)
3. the right to erasure of the data concerning you in accordance with Art. 17 EU GDPR
4. the right to request the restriction of the processing of data in accordance with Art. 18 EU GDPR
5. the right to withdraw your consent at any time without detriment to medical treatment. This does not affect the lawfulness of the processing carried out on the basis of the consent until revocation (Art. 7 para. 3 EU GDPR)
6. the right to object to the future processing of data concerning you in accordance with Art. 21 EU GDPR

You can exercise your rights free of charge and without giving reasons.You also have the right to complain to a data protection supervisory authority about the processing of your personal data by us:

| State Commissioner for Data Protection and Freedom of Information North Rhine-Westphalia  P.O. Box 20 04 44, 40102 Düsseldorf  E-Mail: poststelle@ldi.nrw.de |
| --- |

**Right of withdrawal**

Your consent to this study is voluntary at all times. You can withdraw your consent for the future without giving reasons and thus terminate the personal data collection. You will not suffer any disadvantages as a result of the revocation. Upon request, your personal data can be anonymized or deleted immediately. Of you wish to withdraw your consent, you can contact the study management.

**Thank you for your support!**

**Declaration of consent for participation in the ViSu-study**

**Consent and privacy policy**

I have read the information for patients on the ViSu-study and have understood the aim, procedure and conduct of the study. I have been informed verbally about the nature and significance of the planned participation in the study. I was given sufficient opportunity to clarify any unanswered questions. My consent to participate in this study is voluntary. I have been informed that I can withdraw my consent at any time without giving reasons and without incurring any disadvantages. I have received a copy of the information for patients and this signed declaration of consent. I am aware and agree that personal data about me and saliva samples from me will be collected, stored and analyzed in this study. The use of my personal data is carried out in accordance with the applicable data protection regulations and requires the following voluntary declaration of consent before participation in the study, i.e. without the following consent I cannot participate in the study. I am also aware that I can revoke my consent to the collection, processing and disclosure of my data without giving reasons and without any disadvantage to me with effect for the future. In the event of revocation, I can demand the deletion of all data stored about me up to that point. This is possible until the end of the study, as the anonymized storage does not allow any assignment to my person. I am aware that until the time of revocation, the processing of my data is and remains lawful, and that I have the right to correct my data and to restrict the processing thereof. I am aware that I have the right to receive information about the data stored about me. I also consent to my data being stored and archived for a maximum of 10 years in accordance with the currently valid regulations, after which my data will be deleted.

I hereby voluntarily consent to

- I will participate in the above-mentioned ViSu-study
- personal data will be processed as described and indicated by me
- data will be collected, recorded and stored in paper form or on electronic data carriers of the University Hospital Düsseldorf within the scope of this study
- the saliva samples taken from me will be used exclusively for the determination of cortisol
- the data collected - if necessary - will be pseudonymized (encrypted) and forwarded to a statistician for evaluation and analysis
- I can revoke my consent to the recording, storage and use of my data, whereby my data will be deleted immediately
- that my data will be stored for 10 years after completion or discontinuation of the study and then deleted, provided that there are no legal or statutory retention periods to the contrary

| **I hereby consent to participate in the study an a patient** |  | **I have verbally informed the patient about the nature and significance of the study** |
| --- | --- | --- |
|  |  |  |
| (name of patient) |  | (name of the informing person) |
| (place, date, signature of patient) |  | (place, date, signature) |

**Optional:**

- I would like to be informed and contacted about similar future projects. Please use the following e-mail address:i______________________________________________________
- I would like to be informed about the study results after completion of the study. Please use the following e-mail address: ___________________________________________________
