# Supplementary material for: Virtual Reality and Sound Intervention under Chemotherapy (ViSu): study protocol for a three-arm randomised-controlled trial
Source: BMJ Open. 2025 Apr 9;15(4):e094040. doi: 10.1136/bmjopen-2024-094040 (PMC11987127; doi:10.1136/bmjopen-2024-094040)
Supplement: Supplementary file 6 [file bmjopen-2024-094040.reviewer_comments.pdf]

PEER REVIEW HISTORY

BMJ Open publishes all reviews undertaken for accepted manuscripts. Reviewers are asked to complete a checklist review form and are provided with free text boxes to elaborate on their assessment. These free text comments are reproduced below.

ARTICLE DETAILS

Title (Provisional)

Virtual Reality and Sound Intervention under Chemotherapy (ViSu): study protocol for a three-arm randomized-controlled trial

Authors

Holsteg, Steffen; Ernsten, Luisa; Schaal, Nora; Keßling, Luisa M; Schmutzler, Nele; Fehm, T; Friebe, Verena; Gattermann, Norbert; Ruckhäberle, Eugen; Karger, Andre

---

VERSION 1 - REVIEW

**Reviewer**	**1**
**Name**	**Allan, Stephanie**
**Affiliation**	**University of Glasgow**
**Date**	**30-Nov-2024**
**COI**	**None**

Thank you for inviting me to review this paper. This is a study protocol for a three arm trial which aims to evaluate the feasibility, acceptability and effectiveness of a virtual reality (VR) intervention to reduce psychological distress during chemotherapy. The three study arms being compared are 1) VR mindfulness task, 2) listening to music, and 3) treatment as usual.

This paper is well described. I have focused on suggestions that may enhance clarity for the reader.

Major

1) I do understand word count is always at a premium in BMJ Open. However, I think the "intervention section" on page 5 would be greatly enhanced by a short section describing the theoretical background of mindfulness intervention. By what mechanism is this presumed to work? Do the authors expect creating "presence and immersion" would lead to a sense of distraction from distressing experiences? Given the small word count available, this may be enhanced by a diagrammatic representation.

2) The authors appear interested in the feasibility of implementing VR within this context. Do the authors consider the PSSUQ to provide enough information to make a judgement on this? Typically even in protocol papers there are often "future research" ideas which are useful for science at large in a discussion section. Do the authors have any ideas or views on how we might understanding issues relevant to implementing this intervention more broadly if it is shown to be effective?

3) I did not feel limitations of the study were covered in “Strengths and limitations of this study” on page 2. Would the authors consider reporting some?

Minor

1) Possible typo on page 8, is this meant to be “diverse”? “Patients are asked to indicate their gender (female, male, divers)”. It is great to see information on minority genders being gathered.

**Reviewer**	**2**
**Name**	**Isa, Mohamad Rodi**
**Affiliation**	**Universiti Teknologi Mara Fakulti Perubatan, Public Health Medicine**
**Date**	**11-Dec-2024**
**COI**	**None**

Virtual Reality and Sound Intervention under Chemotherapy (ViSu): A Study Protocol for a three-arm randomized-controlled Trial.

Introduction:
Satisfactory
However, please state more on the justification since it was not very clear and the rationale of doing this study.

Method:

Study design
How open label study can be conducted in single center? How about the contamination issue?
The placebo group will not give anything. Is it ethical not to give anything to the patients?

Intervention:
VR mindfulness intervention s Music
How many times will these interventions be given and what is the interval?
How sure this intervention with certain frequency and time interval can give the actual outcome?
For music overview (appendix A) - who make the selection of the song? Base on which criteria for the selection of those song?

Baseline measures

Generalized Anxiety Disorder - 7 (GAD-7)
How about the validity of this tool?

ASKU
Which version language will be used for this tool?

Please explain what the factorial validity is.

STAI-Trait
Which version language will be used for this tool?
Why only concentrate on Trait anxiety only in baseline measure?
How about the validity of this tool?

Primary outcome measure STAI

- State
Which version language will be used for this tool?
Why only concentrate on Trait state only in baseline measure? How about the validity of this tool?

Secondary outcome measures

VAS
Which version language will be used for this tool? How about the reliability and validity of this tool?

PHQ-4
Which version language will be used for this tool?
What is the meaning of “a factor analysis revealed good fit with 84% of total variance explained”

PA-F-KF
Which version language will be used for this tool?
What is the meaning of “a factor analysis revealed a one-dimensional structure, explaining 42% of the total variance”

MIDDS
Which version language will be used for this tool? How about the validity of this tool?

Heart rate and blood pressure
How will the heart rate and blood pressure be measured?

Saliva cortisol
When will the saliva cortisol be taken?
Cortisol level will be more accurate early in the morning

Sample size
N=82 is for one arm or for whole?

Risk to patients
How do patients monitor the risk after this study finish?

Randomization
Please explain in detail how the randomization done using a computer program into three groups.

Statistical analysis
Please change the word qualitative to categorical
How the decision will be taken to analyse using parametric and non-parametric.

Please change the method of statistical analysis using repeated measures
What is the type of data analysis? - using per-protocol or intention to treat analysis. How about the effect size?

SPIRIT check list

Please register your trial

Please stat the strategy to improve effectiveness to intervention protocol in the test. Please state the post-trial care to the patietns in the text.

**Reviewer**	**3**
**Name**	**Sato, Daisuke**
**Affiliation**	**Chiba University Graduate School of Medicine School of Medicine, Cognitive Behavioral Physiology**
**Date**	**16-Dec-2024**
**COI**	**None**

The study deals with the effectiveness of VR and music interventions during the application of chemotherapy.

The authors should also clarify the points listed below.

1. Inclusion criteria

Please explain why the authors do not set a lower or upper age limit.

2. Exclusion criteria

Authors should describe how they determine the severity of visual and hearing impairment.

3. Statistical Analysis

The authors should define the statistical analysis subjects.

These comments will be helpful.

VERSION 1 - AUTHOR RESPONSE

Reviewer 1
Ms. Stephanie Allan, University of Glasgow

Comments to the Author:
Thank you for inviting me to review this paper. This is a study protocol for a three arm trial which aims to evaluate the feasibility, acceptability and effectiveness of a virtual reality (VR) intervention to reduce psychological distress during chemotherapy. The three study arms being compared are 1) VR mindfulness task, 2) listening to music, and 3) treatment as usual.

This paper is well described. I have focused on suggestions that may enhance clarity for the reader.

Major

1. I do understand word count is always at a premium in BMJ Open. However, I think the "intervention section" on page 5 would be greatly enhanced by a short section describing the theoretical background of mindfulness intervention. By what mechanism is this presumed to work? Do the authors expect creating "presence and immersion" would lead to a sense of distraction from distressing experiences? Given the small word count available, this may be enhanced by a diagrammatic representation.

Author response: Thank you for this very helpful comment. We completely agree with you and have added a paragraph to the intervention section briefly explaining what mindfulness is, how mindfulness can be developed as a skill, the mechanisms of action involved and the possibilities of implementing it in VR. Due to space limitations, we did not discuss this in more detail, but the section does give the reader more information about why this intervention was chosen.

"Mindfulness, as defined by Kabat-Zinn [22], refers to the intentional self-regulation of attention to the present moment without judgment. To cultivate mindfulness skills, these practices are typically embedded in meditation exercises [22]. Over time, this concept has been integrated into psychological interventions and has evolved into a variety of meditation practices, many of which have been tested in randomized controlled trials [23]. Such exercises involve directing attention to thoughts, emotions, and bodily sensations, simply observing them as they arise and pass away [24]. Potential mechanisms underlying the effects of mindfulness include attention and emotion regulation, increased body awareness, and a shift in perspective on the self [24]. VR may offer several advantages for mindfulness meditation. By shielding users from distracting environmental factors that might otherwise interfere with meditation, VR can create a more focused and immersive experience [14, 15]. The sense of presence that arises during VR exercises is described as engaging and is considered to enhance mindfulness [14]. The combination of audiovisual stimuli in VR may further reduce mind-wandering by anchoring attention [14, 25]." (p. 5-6)

2. The authors appear interested in the feasibility of implementing VR within this context. Do the authors consider the PSSUQ to provide enough information to make a judgement on this? Typically even in protocol papers there are often "future research" ideas which are useful for science at large in a discussion section. Do the authors have any ideas or views on how we might understanding issues relevant to implementing this intervention more broadly if it is shown to be effective?

Author response: Thank you for bringing this to our attention. We appreciate the opportunity to clarify how we plan to assess feasibility. In addition to the PSSUQ, we will examine screening and recruitment numbers to gain insights into which patients choose to participate in the study and the reasons for non-participation (see section Baseline Measures, Screening, p. 8). Furthermore, feasibility will be assessed based on documentation from the study staff during interviews, reasons for dropout, and possible side effects.

Beyond usability (PSSUQ), the evaluation questionnaire includes additional self-developed items capturing various dimensions. The number of items varies slightly depending on the experimental condition, with specific questions tailored to the VR and music interventions.

Additionally, free-text responses allow participants to provide suggestions for improvement and highlight potential challenges. A full version of the questionnaire can be found in *Appendix D*, and further details are provided in the *Secondary Outcome Measures* section (p. 10).

This feasibility data will be used to discuss the issues you have raised. For example, whether outpatient chemotherapy is the right place to offer a VR intervention, how much capacity is needed for the recruitment and delivery aspect outside of a trial, and what forms of support patients find helpful. A publication is planned to discuss this aspect in the context of the trial data and experience.

To enhance clarity regarding our feasibility assessment, we have revised the *Statistical Analysis* section (p. 12) accordingly.

"The feasibility of the intervention will also be evaluated based on multiple data sources. A descriptive analysis of the screening and recruitment data will provide insights into patient participation and reasons for non-participation. Additionally,
feasibility will be assessed based on study staff documentation during data collection sessions, as well as recorded reasons for dropout, and potential side effects.
Furthermore, the evaluation questionnaire, which includes both usability assessment and self-developed items, will be analyzed. The participants' comments in the free text fields of the evaluation questionnaire will be categorized based on their content into issues and suggestions for improvement." (p. 13)

3. I did not feel limitations of the study were covered in "Strengths and limitations of this study" on page 2. Would the authors consider reporting some?

Author response: Thank you for pointing this out. As described earlier in the editor's comments, we have now added a limitation to the *Strengths and limitations* section.

"- Limited standardization of the assessment environment, making it challenging to control for potential confounding variables during data collection." (p. 2)

Minor

1. Possible typo on page 8, is this meant to be "diverse"? "Patients are asked to indicate their gender (female, male, divers)". It is great to see information on minority genders being gathered.

Author response: Thank you, it was indeed a typo. We changed it to "diverse".

Reviewer 2

Prof. Mohamad Rodi Isa, Universiti Teknologi Mara Fakulti Perubatan

Comments to the Author:
Good manuscript but need to do some corrections (see attached PDF).

Introduction:
Satisfactory

However, please state more on the justification since it was not very clear and the rationale of doing this study.

Author response: Thank you for your feedback. We have edited the introduction in several places to make the rationale for the study more explicit (see p. 4).

Study design

How open label study can be conducted in single center? How about the contamination issue? The placebo group will not give anything. Is it ethical not to give anything to the patients?

Author response: Thank you for your valuable comments. This study is conducted as an open-label trial at a single center to assess the feasibility and potential effects of the interventions before considering a larger multicenter trial. While we acknowledge the potential risk of contamination in a single-center setting, we have taken measures to minimize this risk. Study personnel follow strict standardized protocols to ensure consistent intervention delivery and avoid cross-group influence. Importantly, study participants had no direct contact with each other, and data collection sessions were conducted individually, not simultaneously.

The control group will not receive any additional interventions beyond standard medical care. The inclusion of a control group without additional interventions is ethically justified, as the potential superiority of the interventions over standard care in this specific setting has not yet been clearly established. This exploratory investigation aims to assess whether the interventions have meaningful effects under these conditions. All participants continue their standard oncological treatment and are referred to available psycho-oncological support services if they express psychological distress, ensuring that all patients receive appropriate care while allowing for a meaningful evaluation of the interventions.

Intervention:

VR mindfulness intervention & Music

How many times will these interventions be given and what is the interval? How sure this intervention with certain frequency and time interval can give the actual outcome?

Author response: In our study, VR and music intervention will take place on two consecutive chemotherapy sessions. In choosing the length and interval of the intervention, we were guided by the preliminary work of, for example, Chirico et al. (2020), who also used a 20-minute VR intervention. We have added an explanation for the usefulness of mindfulness and potential benefits of presenting mindfulness-based intervention via VR (Interventions, p. 4), which will hopefully give more detail about the underlying assumption of the effectiveness.

For music overview (appendix A) – who make the selection of the song? Base on which criteria for the selection of those song?

Author response: A music psychologist generated the playlists based on Nilsson's specifications (instrumental tracks, varying between 60 and 80 beats per minute). We have added this information to the music intervention section. These playlists have been used in other studies by our team; the evaluation did not result in any comments on the music titles.

“The playlists consist of music titles selected by a music psychologist according to the recommendations of Nilsson [30] and used in a previous project of our group [31].” (p. 6)

Baseline measures

Generalized Anxiety Disorder - 7 (GAD-7)

How about the validity of this tool?

ASKU

Which version language will be used for this tool?

Please explain what the factorial validity is.

STAI-Trait

Which version language will be used for this tool?

Why only concentrate on Trait anxiety only in baseline measure?

How about the validity of this tool?

Primary outcome measure

STAI - State

Which version language will be used for this tool?

Why only concentrate on Trait state only in baseline measure?

How about the validity of this tool?

Author response: As the “STAI trait” assesses anxiety as a more stable personality trait (Grimm, 2010; Laux et al., 1981; Spielberger et al. 1983), we only assess it as a baseline measure. It can be expected that answers given in the „STAI trait“ will not change to a greater extent over the course of study participation.

Secondary outcome measures

VAS

Which version language will be used for this tool?

How about the reliability and validity of this tool?

PHQ-4

Which version language will be used for this tool?

What is the meaning of “a factor analysis revealed good fit with 84% of total variance explained”

PA-F-KF

Which version language will be used for this tool?

What is the meaning of “a factor analysis revealed a one-dimensional structure, explaining 42% of the total variance”

MIDDS

Which version language will be used for this tool?

How about the validity of this tool?

Author response: Thank you for the comments concerning all measures that were used in our study. Since most questions focused on the language used and issues of validity and reliability, we have decided to address them in a summarized form. All questionnaires were administered in German. We have added this to the text (Measures section) as well.

"All questionnaires were used in the German version." (p. 7)

Furthermore, German validation studies were used to describe reliability and validity. In this context, we closely followed the information provided in the respective validation studies (i.e., Cronbach's α, McDonald's ω). However, this also means that we have provided different measures of reliability and validity for the different measures, depending on the data available.

For visual analogue scales, it is more complicated to report a validity and reliability, however, Wewers and Lowe (1990) reviewed different approaches to use visual analogue scales for the assessment of pain and anxiety. We also cite this report in our manuscript. Where applicable, we have included information about the factorial structure of the questionnaires (e.g. „a factor analysis revealed a one-dimensional structure, explaining 42% of the total variance"), which refers to the construct validity of the measurement. For the MIDOS we have reported the following: „The questionnaire reveals good psychometric properties, with internal consistencies varying between α = .67 and .73 and the test-retest variability varying between r = .69 and r = .57 [39]." (p 10).

Heart rate and blood pressure
How will the heart rate and blood pressure be measured?

Author response: Thank you for your valuable comment. To ensure transparency, we have revised the text to provide additional details on the measurement process. Heart rate and blood pressure are recorded continuously in 5-minute intervals using a pulse oximeter and blood pressure monitor. These values are actively collected by a member of the study team, who is present throughout the intervention. This clarification has now been added to the manuscript:

"Heart rate and blood pressure are measured continuously from the beginning to the end of the intervention in 5-minute intervals in beats per minute (bpm) using a pulse oximeter and blood pressure monitor. A member of the study team records these values while accompanying the intervention." (p. 10)

Saliva cortisol
When will the saliva cortisol be taken?
Cortisol level will be more accurate early in the morning

Author response: We will assess saliva samples "before the beginning of the intervention and 25 minutes after the end of the intervention" (*Methods* section, p. 10) for both consecutive chemotherapy sessions. There are different approaches for the assessment and applicability of cortisol levels using saliva samples. As you suggested, the cortisol awakening response (CAR) is hypothesized to be an anticipatory reaction of the body. However, in our study, we are interested in changes in hypothalamic-pituitary-adrenal axis activity that may

be linked to our interventions. Therefore, we chose the approach as described in our manuscript.

Sample size

N=82 is for one arm or for whole?

Author response: The *N* = 82 refers to the total sample.

Risk to patients

How do patients monitor the risk after this study finish?

Author response: Thank you for your helpful comment. We are pleased to provide further details regarding post-trial care. Patients remain under the ongoing care of their treating physicians who recommended or approved their participation in the study, where potential side effects can still be discussed. In addition, short-term access to psycho-oncological support will be provided through the department of psychosomatic medicine and psychotherapy for patients who express a need for psychological support after study completion. This has now been included in the 'Risk to patients' section.

“Patients will continue to receive ongoing medical care after the end of the trial, where potential side effects can still be discussed. In addition, short-term access to psycho- oncological support will be provided through the department of psychosomatic medicine and psychotherapy for patients who express a need for psychological support after study completion.” (p. 12)

Randomization

Please explain in detail how the randomization done using a computer program into three groups.

Author response: Thank you for pointing this out. We have tried to provide more information to explain the randomization process in more detail. We included the command we used in Excel and made it clear that the patients were equally divided between the three groups. We hope this makes the process clearer.

“Randomization is conducted using random numbers. Using a computer program (Microsoft Excel, Microsoft Corporation), a team member who does not participate in recruiting patients generates an excel sheet with numbers representing the three intervention groups (using the command "=RANDBETWEEN(1,3)"). The random allocation sequence assigns patients in equal proportions to one of the three intervention groups.
In this sheet, the numbers are masked until a new patient is enrolled, which assures that members of the study team accountable for participant recruitment are blinded until patients’ consent.” (p. 13)

Statistical analysis

Please change the word qualitative to categorical

Author response: Thank you for this helpful comment. The term *qualitative* was originally used because the free-text responses provide open-ended feedback. However, we agree that *categorical* is a more precise term in this context, as we plan to systematically classify

the content of the responses into categories such as "problems" and "suggestions for improvement." We have updated the text accordingly to reflect this adjustment.

How the decision will be taken to analyse using parametric and non-parametric.

Author response: Thank you for pointing out the lack of clarity in our description of the statistical analysis. We have revised the text to ensure greater precision.

"Parametric analysis methods are used to evaluate the primary and secondary target variables. If assumptions for parametric tests are violated, appropriate non-parametric alternatives will be considered." (p. 13)

Given our study design and sample size, we have chosen ANOVA as it best captures our mixed factorial design with both between- and within-subject factors. ANOVA is generally robust to violations of normality, particularly when group sizes are balanced. However, if key assumptions - such as normality of residuals (assessed via Shapiro-Wilk test) or homogeneity of variances (Levene's test) - are substantially violated, we will discuss the use of non-parametric alternatives. Potential alternatives include the Kruskal-Wallis test for between-group comparisons and Wilcoxon signed-rank tests for within-subject comparisons, though these methods do not fully account for interaction effects.

Since these adjustments will only be necessary if assumptions are violated, we have not preemptively included them in the main text but will transparently report any deviations and methodological adaptations in the final analysis.

Please change the method of statistical analysis using repeated measures

Author response: Thank you for your comment about the statistical analysis. We acknowledge that our design includes a repeated measures component as we are assessing changes from pre to post. However, we prefer to retain the term "mixed factorial ANOVA" because our primary focus is not only on the within-subject factor (time: pre vs. post), but also on the between-subject factor (intervention group: VR, music, control). The expected interaction between these factors is central to our analysis.

What is the type of data analysis? - using per-protocol or intention to treat analysis.
How about the effect size?

Author response: Thanks for pointing this out. We have added information about the analysis and effect size in the text.
We will conduct both an Intention-to-Treat analysis and a Per-Protocol analysis to ensure the robustness of our findings. Any differences in outcomes between these approaches will be reported and discussed in the results section. The ITT provides a more realistic estimate of effectiveness in clinical practice (interesting in terms of feasibility), while PP reflects the treatment's efficacy under ideal conditions (Moler-Calafell et al., 2024).

"Situational anxiety as the primary outcome (measured using the STAI state) is analyzed using separate 3x2 mixed factorial ANOVAs for each of the two chemotherapy sessions, with the between-subject factor "intervention" (virtual reality vs. music vs. control) and

the within-subject factors "time" (before vs. after intervention). A probability of error of α=.05 is set. The unit of analysis is individual patients. Effect sizes will be reported using partial eta squared (η^2) for ANOVA analyses. Cohen's d is calculated for post-hoc comparisons when appropriate. The secondary outcome criteria are analyzed exploratively, also using ANOVAs and post hoc tests as appropriate. There is no adjustment of the probability of error. The statistical analysis is carried out with the help of standard statistical software (e.g., R, SAS, SPSS). Both an intention-to-treat analysis and a per-protocol analysis will be performed. The results will be compared and discussed." (p. 13)

SPIRIT check list

Please register your trial

Author response: We have registered with the German Clinical Trials Register (DRKS), as stated on page 2. The DRKS is recognized as the WHO primary registry for clinical trials in Germany and meets the requirements of the ICMJE.
"Trial Registration: German Clinical Trials Register (DRKS) - ID: DRKS00029738, registered August 16th, 2022". (p. 2)

Please stat the strategy to improve effectiveness to intervention protocol in the test.

Author response: Thank you for your helpful comment. We acknowledge the importance of implementing strategies to improve the effectiveness of the intervention protocol. To address this, we have now included a specific section "Implementation and quality assurance of the intervention" in the Intervention section of the manuscript that outlines the actions taken to ensure protocol fidelity and effectiveness.

"***Implementation and quality assurance of the intervention***
To optimize intervention effectiveness, several strategies are being implemented. Recruitment data, reasons for non-participation (via a screening questionnaire), and dropout rates are systematically documented to assess feasibility. Following recruitment, participants receive standardized instructions for correct VR and music intervention application. Additionally, study staff are trained to provide consistent guidance and address technical difficulties.
To further ensure procedural consistency, a structured documentation form includes a checklist for standardization and records start/end times and disruptions. Noise- canceling headphones are used to minimize external distractions, ensuring participants can fully engage in the intervention.
Furthermore, weekly team meetings facilitate continuous process evaluation, allowing for structured feedback on data collection, recruitment challenges, and potential implementation barriers.
At the end of the study, feasibility and user experience data, including responses from an evaluation questionnaire, will be analyzed to identify challenges and potential areas for improvement."

Please state the post-trial care to the patietns in the text.

Author response: Thank you for your suggestion. We acknowledge the importance of clearly stating the post-trial care for participants. In our study, participation in the intervention does not replace standard medical or psychological care, and patients continue to receive their usual oncological treatment with their physicians, who recommended or approved their participation in the study. We have now included the post-trial care information in the

manuscript to clarify the procedures.

"Patients will continue to receive ongoing medical care after the end of the trial, where potential side effects can still be discussed. In addition, short-term access to psycho-oncological support will be provided through the department of psychosomatic medicine and psychotherapy for patients who express a need for psychological support after study completion." (p. 12)

Reviewer 3

Dr. Daisuke Sato, Chiba University Graduate School of Medicine School of Medicine

Comments to the Author:
The study deals with the effectiveness of VR and music interventions during the application of chemotherapy. The authors should also clarify the points listed below.

1. Inclusion criteria

Please explain why the authors do not set a lower or upper age limit.

Author response: Thank you for your helpful suggestion. We did indeed forget to mention that we only include patients aged 18 and over. We have now added this to the sample section. We did not specify an upper age limit because we did not want to narrow down the population any further and because we wanted to see which patients would be willing to participate in the study in terms of feasibility.

"All patients are pre-screened for eligibility by their attending physician. The eligibility criteria for the study include a) the general physical condition of the patient, *b) an age of 18 years or older*, […]" (p. 5)

2. Exclusion criteria

Authors should describe how they determine the severity of visual and hearing impairment.

Author response: We collect information about severe visual and hearing impairments from the patients themselves. We ask them if they have any problems listening to music or using the VR glasses. Mild hearing impairment can be compensated by adjusting the volume, and the patient's glasses can be worn under the VR headset. In addition, a member of the study team will be present throughout the session to help adjust the VR glasses and document any problems.

"Patients are not eligible for the study if they have a) severe visual and/or hearing impairment (self-reported), […]" (p. 5)

3. Statistical Analysis

The authors should define the statistical analysis subjects.

Author response: Thank you for this valuable suggestion. We have added further details to clarify the analysis. The unit of analysis is the individual patient. In addition, based on feedback from the other reviewers, we have added further information on the statistical analysis to more clearly define the approach.

"Situational anxiety as the primary outcome (measured using the STAI state) is analyzed using separate 3x2 mixed factorial ANOVAs for each of the two chemotherapy sessions, with the between-subject factor "intervention" (virtual reality vs.

music vs. control) and the within-subject factors "time" (before vs. after intervention). A probability of error of α=.05 is set. The unit of analysis is individual patients. Effect sizes will be reported using partial eta squared (η^2) for ANOVA analyses. Cohen's d is calculated for post-hoc comparisons when appropriate. The secondary outcome criteria are analyzed exploratively, also using ANOVAs and post hoc tests as appropriate. There is no adjustment of the probability of error. The statistical analysis is carried out with the help of standard statistical software (e.g., R, SAS, SPSS). Both an intention-to-treat analysis and a per-protocol analysis will be performed. The results will be compared and discussed." (p.13)

References:

Chirico, A., Maiorano, P., Indovina, P., Milanese, C., Giordano, G. G., Alivernini, F., ... & Giordano, A. (2020). Virtual reality and music therapy as distraction interventions to alleviate anxiety and improve mood states in breast cancer patients during chemotherapy. *Journal of cellular physiology*, *235*(6), 5353-5362.
Grimm J. State-Trait-Anxiety Inventory nach Spielberger: Deutsche Lang- und Kurzversion. Methodenforum der Universität Wien. MF-Working Paper. 2009;2.
Laux L, Glanzmann P, Schaffner P, Spielberger CD. Das State-Trait-Angstinventar. Weinheim: Beltz; 1981.
Molero-Calafell, J., Burón, A., Castells, X., & Porta, M. (2024). Intention to treat and per protocol analyses: differences and similarities. *Journal of Clinical Epidemiology*, 173, 111457.
Spielberger, C. D., Gorsuch, R. L., Lushene, R., Vagg, P. R., & Jacobs, G. A. (1983). Manual for the State-Trait Anxiety Inventory. Palo Alto, CA: Consulting Psychologists Press.

VERSION 2 - REVIEW

Reviewer 1
Name Allan, Stephanie
Affiliation University of Glasgow
Date 06-Mar-2025
COI

I think the comments I suggested have been well addressed.

Reviewer 3
Name Sato, Daisuke
Affiliation Chiba University Graduate School of Medicine School of Medicine, Cognitive Behavioral Physiology
Date 21-Feb-2025
COI

The manuscript has been much improved, but I need some clarification. Please share my arguments below.

1. Please define the subjects for statistical analysis based on the frequency and duration of receiving the intervention.

Is a subject not considered a dropout if he/she receives the intervention for even one minute?

2. Please specify the name and version of the software for statistical analysis.

These comments will be helpful.

VERSION 2 - AUTHOR RESPONSE

Reviewer 1

Ms. Stephanie Allan, University of Glasgow

Comments to the Author:
I think the comments I suggested have been well addressed.

Reviewer 3

Dr. Daisuke Sato, Chiba University Graduate School of Medicine School of Medicine

The manuscript has been much improved, but I need some clarification. Please share my arguments below.

1. Please define the subjects for statistical analysis based on the frequency and duration of receiving the intervention.
   Is a subject not considered a dropout if he/she receives the intervention for even one minute?

Author response: Thank you for your valuable comment and for helping us improve the clarity of our manuscript. We have now explicitly defined the criteria for including subjects in the statistical analysis based on the frequency and duration of the received interventions.

> "Participants will be included in the statistical analysis if they have completed both assessment time points and received the intervention for a minimum of 10 minutes per session.
> Furthermore, the intervention must be administered in consecutive sessions as scheduled. Participants who do not meet these criteria will be classified as dropouts." (p. 13)

2. Please specify the name and version of the software for statistical analysis.

Author response: We will use SPSS 29 and have now indicated this in the manuscript.

> "The statistical analysis is carried out with the help of standard statistical software (SPSS 29)." (p. 13)
